# Molecular surveillance and genetic diversity of *Anaplasma* spp. in cattle (*Bos taurus*) and goat (*Capra aegagrus hircus*) from Hainan island/province, China

**DOI:** 10.1186/s12917-023-03766-2

**Published:** 2023-10-18

**Authors:** Sa Zhou, Liangyuan Huang, Yang Lin, Biswajit Bhowmick, Jianguo Zhao, Chenghong Liao, Qingfeng Guan, Jinhua Wang, Qian Han

**Affiliations:** 1 One Health Institute, Hainan University, Haikou, 570228 Hainan China; 2 College of Animal Science and Technology, Hainan University, Haikou, 570228 Hainan China; 3 Laboratory of Tropical Veterinary Medicine and Vector Biology, School of Life Sciences, Hainan University, Haikou, 570228 Hainan China

**Keywords:** Ruminants, Nested PCR, Hainan, *Anaplasma*, Co-infection

## Abstract

Anaplasmosis is a highly prevalent tick-borne intracellular bacterial disease that affects various host species globally, particularly ruminants in tropical and subtropical regions. However, information regarding the distribution and epidemiology of anaplasmosis in small and large ruminants on Hainan Isalnd is limited. To address this knowledge gap, the present study aimed to assess the occurrence of *Anaplasma* spp. infections in goats (*N* = 731) and cattle (*N* = 176) blood samples using nested PCR and conventional PCR based assays. The results revealed an overall prevalence of 30.1% in goats and 14.8% in cattle. The infection rates of *A. bovis*, *A. phagocytophilum*, *A. ovis* and *A. capra* in goat samples were 22.7%, 13.8%, 2.0% and 3.4%, respectively, while the infection rates of *A. bovis*, *A. phagocytophilum* and *A. marginale* in cattle samples were 11.4%, 6.3% and 5.7%, respectively. *A. bovis* exhibited the highest prevalence among the Anaplasma spp. in both goat and cattle samples. In addition, the most frequent co-infection was the one with *A. phagocytophilum* and *A. bovis.* It was found that the age, sex and feeding habits of cattle and goats were considered to be important risk factors. Evaluation of the risk factor relating to the rearing system showed that the infection rate for the free-range goats and cattle was significantly higher when compared with stall-feeding system.

This study represents one of the largest investigations on the distribution, prevalence, and risk factors associated with *Anaplasma* infection in ruminants on Hainan Island, highlighting a higher circulation of the infection in the region than previously anticipated. Further reasesrch is necessary to investigate tick vectors, reservoir animals, and the zoonotic potential of the *Anaplasma* spp. in this endemic region of Hainan Island.

## Background

Anaplasmosis is a tick-borne disease caused by a group of obligate intracellular bacteria of the genus *Anaplasma*. It has a worldwide distribution, particularly in tropical, subtropical and temperate regions, and is considered one of the most important pathogens in humans and animals. *Anaplasma* belongs to the family of *Anaplasmataceae*, order *Rickettsiales*. To date, seven *Anaplasma* spp. are most commonly reported in domestic animals: *A. phagocytophilum*, *A. ovis*, *A. bovis*, *A. marginale*, *A. centrale*, *A. platys* and *A. capra* [1].

*A. phagocytophilum* is a zoonotic pathogen that mainly infects human and animal neutrophils, It can cause an acute febrile illness known as human granulocytic anaplasmosis (HGA) [2]. The first human case of HGA was detected in the United States in 1990, and several cases have since been reported in countries in Europe and Asia. In China, the first case of HGA was detected in Anhui Province in 2006, and then the prevalence of HGA was found in several provinces and regions in China. Anaplasmosis generally presents with nonspecific symptoms such as fever, chills, malaise, headache, and myalgias. In severe cases, multiple organ function is impaired, and death can occurs [3]. The reservoir for *A. phagocytophilum* includes domestic and wild mammals, such as sheep, goat, cow, horse, deer, cat, dog and human [4–9].

*A. ovis*, which has the the same affinity to host cells as *A. marginale* and *A. centrale*, is a tick-borne obligate intraerythrocytic bacterium that commonly infects camels, sheep, goats, cattle and wild ruminants [6, 10]. Additionally, it was detected in humans in Cyprus in 2006. Clinical manifestations such as fever, fatigue, loss of appetite, reduced milk production miscarriage and Lower mortality rates are common in ruminants with ovine anaplasmosis infections [11]. Goats are more prone to outbreaks than sheep [12]. Moreover, for other anaplasmas, *A. ovis* infection may predispose to other microbial or parasitic infections, leading to the exacerbation of clinical symptoms and eventual death [13].

*A. marginale*, which mainly infects cattle, has been increasingly detected in other animal species, horse and asses [14]. It is the most pathogenic among several *Anaplasma* spp*.*for cattle. The pathogen has been recorded on several continents, including South America [15–18], Latin America [19, 20], Africa [21–24], Asia [18, 25–28], Australia [24] and Europe [29]. However, in China, the reports of marginal apocrysis are very rare in China. *A.centrale* is closely related to *A. marginale* and is considered a subspecies of *A. marginale*, causing only mild anaemia in animals [30]. It is used as a live vaccine against the *A. marginale* in Africa, Australia, Latin America, and Israel [13].

*A. bovis* is an obligate parasitic pathogen in monocytes. *A. bovis* infection causes anaplasmosis in cattle, presenting a variety of clinical symptoms, including fever, weight loss, and lower milk production. In the acute phase of the disease, it occasionally causes abortion and death [31]. However, most of infected animals are asymptomatic. Besides the aforementioned ruminants, DNA of *A. bovis* has also been detected in goats, sheep, dogs, wild cats, swine, as well as monkeys worldwide [32–35].

*A. capra*, an emerging zoonotic *Anaplasma*, was first discovered in asymptomatic goats in China [36]. Soon after, 28 out of 477 hospital patients with a history of tick bites in Heilongjiang, China, were infected with *A. capra* [1]. The disease is widely distributed in several provinces in China and mainly affects sheep, goats and humans. In Liaoning, Henan, and Heilongjiang provinces of China, sheep infected with *A. capra* were detected [37]. The disease was also detected in goats in seven Chinese provinces/autonomous region: Guizhou, Henan, Inner Mongolia, Shanxi, Xinjiang, Yunnan and Gansu [38]. A new species with zoonotic potential, known as *A*. *capra*, has also been discovered in various hosts across European and Asian countries. The presence of *A*. *capra* has also been documented in domestic ruminants in Türkiye [39]. In addition to sheep, goats and humans, the pathogen also appears to have a wide range of hosts around the world, including cattle [40] and water deer [41] in Korea, deer [42] in France, water buffalo [43] in Türkiye, and cattle [44] in Kyrgyzstan.

*A. platys*, the agent of infectious canine cyclic thrombocytopenia (ICCT), is a bacterium parasitized in the canine platelets [21]. Additionally, two clinical cases with single *A. platys* infection were recently confirmed in two women from Venezuela [45]. These findings further indicate that, in addition to *A. phagocytophilum* being recognized as zoonotic, *A. ovis, A. capra* and *A. platys* are also potential zoonotic pathogens, which can be easily ignored.

There have been many reports about *Anaplasma* spp. in the central and northern regions of China [46–52]. However, there have been few studies on *Anaplasma* infections in southern China. Considering the scarce data regarding the epidemiology of *Anaplasma* in ruminants in Hainan, China, we conducted a survey in Hainan island/province to obtain this needed information. The aim of this study is to determine the species of *Anaplasma* in ruminants, and to evaluate the prevalence of *Anaplasma* in Hainan.

## Materials and methods

### Study area

**Fig. 1:**
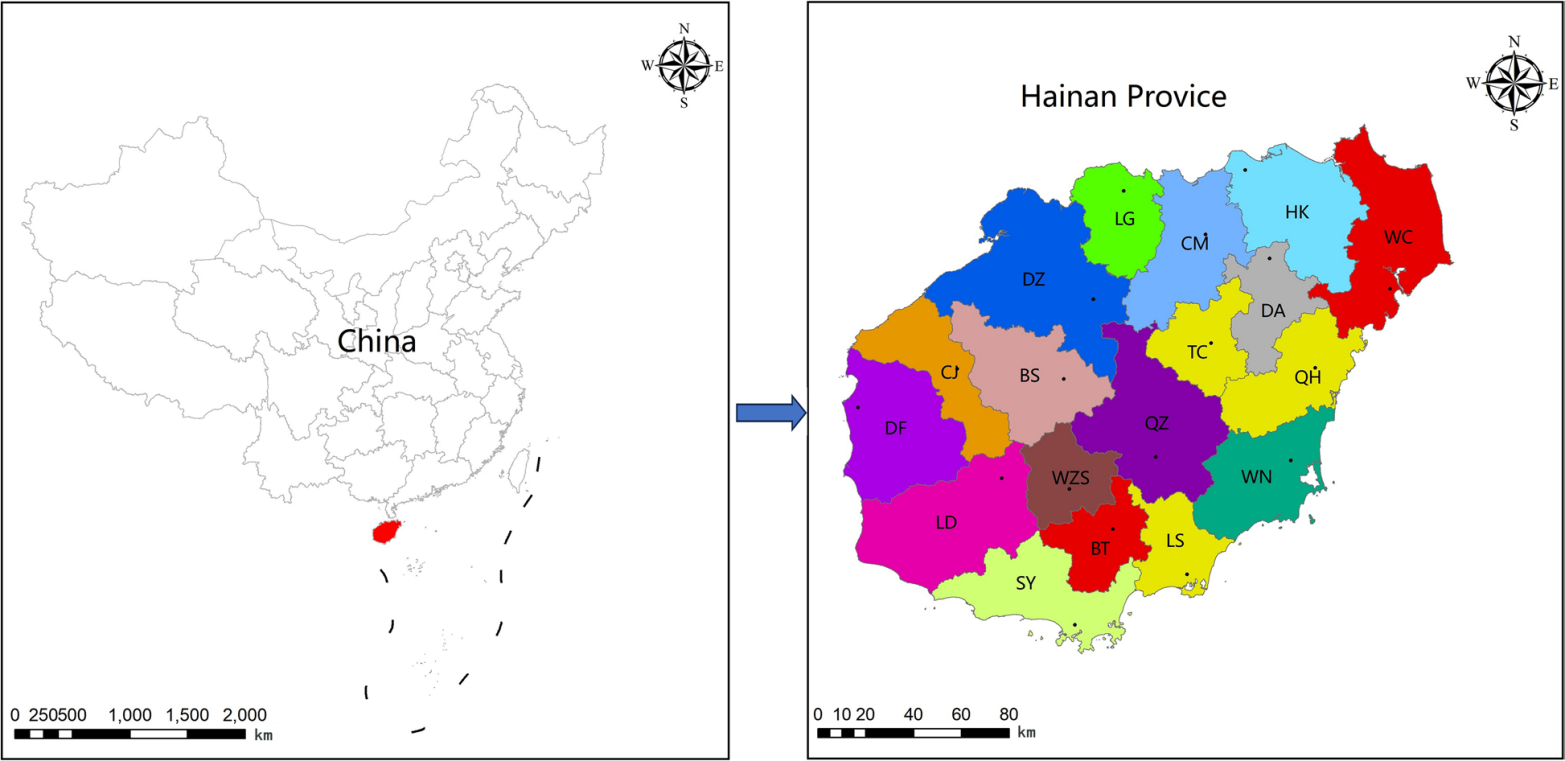
The geographical location of the study area (Hainan Province, China) and the distribution sites of different cities and counties. QZ: Qiongzhong, DF: Dongfang, WC: Wenchang, CJ: Changjing, DZ: Danzhou, DA: Dingan, WZS: Wuzhishan, QH: Qionghai, LS: Lingshui, BS: Baisha, CM: Chengmai, BT: Baoting, WN: Wanning, LD: Ledong, TC: Tunchang, LG: Lingao, SY: Shanya

This study was conducted in Hainan province (also called Hainan Island), which is located in the South China sea (between 108° 37’ and 111° 03’ E longitude and 18° 10’ and 20° 10’ N latitude) (Fig. 1). This part of the island is renowned for its tropical climate, which is completely different from that of the Chinese mainland. It encompasses 35,400 km^2^ of land, with an average rainfall between 1000–2600 mm/year (occurring mostly from July to October) and an average annual temperature of 26.5 °C.

### Blood sample collection

Between June 2018 and August 2020, whole blood samples were collected from the jugular vein of goats and cattle using EDTA Vacutainer tubes. A total of 731 goat samples were randomly collected from 18 counties in Hainan province, of which 122 were male and 609 were female. Secondly, blood samples were collected from a total of 176 individual cattle from 4 counties, of which 155 were female and 21 were male. The blood samples were stored at − 20 °C until molecular analyses. The sampling locations are shown in Fig. 1. Additionally, the feeding system, sex, and age were recorded. Age was measured according to dentition; goats younger than 1.5 years were considered as young and goats older than 1.5 years were considered to be adults. For cattle, those younger than 3 years were considered young, and those older than 3 years were considered adults. Stall-feeding livestock farming is a system in which animals were well maintained and fed in stall with limited access to land. All procedures to gather samples from animals were approved by Hainan University Institutional Animal Care and Use Committee (HNUAUCC-2019-0000A).

### Extraction of whole blood DNA

Frozen EDTA blood samples were thawed and vortexed at room temperature. Using the Blood Genomic DNA Extraction Kit (Sangon Biotech, Shanghai, China), the blood genome extraction procedure was performed according to the manufacturer's instructions, 200 μL of whole blood was aspirated for DNA extraction, and 50 μL of purified DNA was obtained. DNA concentration (ng/μL) and purity were measured using a Nanodrop ND1000 spectrophotometer (Thermo Scientific©, USA) to measure DNA concentration (ng/μL) and purity.DNA samples with a concentartion less than 5 ng/μL were discarded, and whole blood DNA was re-extracted. The DNA was stored at -20 °C.

### Nested PCR and single PCR amplification

The primer sequences used in this study are presented in Table 1. For *A. phagocytophilum* and *A. bovis*, nested PCR was carried out to amplify the *16S rRNA* gene as described previously [53]. The first round of amplification primers were *EE1* and *EE2* [54]. During the second round, *A. bovis* specific primers AB1f and *AB1r* were used that generate a product of 551 bp, and the *A. phagocytophilum* specific primers *SSAP2f* and *SSAP2r* were used to generate a product of 641 bp [53, 55, 56]. For the *A. ovis* and *A. marginale*, the partial segment of *MSP4* gene was amplified using primers *MSP4f* and *MSP4r* [56]. Based on previously published data, PCR was performed using primers *groELf* and *groELr* to amplify partial *groEL* gene of *A. capra* [57]. All primers mentioned above were synthesized by Shanghai Bioengineering Co. The first round PCR reactions were performed in a final volume of 25 μL containing 13μL Mix enzyme (Novozymes, Nanjing, China) 10 μL of nuclease free water, 0.5 μL of each primer and 1 μL of DNA. Thermal cycling system was as described in a previous report [58] for *A. phagocytophilum* and *A. bovis*. The final volume of the second PCR reaction was 50 μL containing 25 μL of LMaxase (Novozymes, Nanjing, China), 21 μL of nuclease free water, 1 μL of each primer and 2 μL of the product of the first PCR reaction. the thermal cycling system was as described in a previous report [55]. For *A. ovis*, *A. marginale* and *A. capra*, the final system of the reaction was 25 μL. thermal cycling reaction conditions were as in Table 2. The PCR products were electrophoresed in a 1.5% agarose gel. The electrophoresis was also performed at a constant voltage of 120 V. When the electrophoretic bands ran to the appropriate position, the electrophoresis instrument was turned off, the agarose gel was removed and placed under a gel imaging UV light source to observe the results and photographed.

**Table 1:** Primers used for the amplification of *16S rRNA, MSP4* and *Gro EL*

Primer	Sequence 5’ to 3’	Assay	Target gene	Amplicon size (bp)	References
EE-1	TCCTGGCTCAGAACGAACGCTGGCGGC	*Anaplasma*	*16S rRNA*	1433	Barlough et al., 1996 [54]
EE-2	AGTCACTGACCCAACCTTAAATGGCTG				
SSAP2f	GCTGAATGTGGGGATAATTTAT	A. *phagocytophilum*	*16S rRNA*	641	Kawahara et al., 2006 [55]
SSAP2r	ATGGCTGCTTCCTTTCGGTTA				
AB1f	CTCGTAGCTTGCTATGAGAAC	*A. bovis*	*16S rRNA*	551	Kawahara et al., 2006 [55]
AB1r	TCTCCCGGACTCCAGTCTG				
AC1f	CTGCTTTTAATACTGCAGGACTA	*A.centrale*	*16S rRNA*	426	Kawahara et al., 2006 [55]
AC1r	ATGCAGCACCTGTGTGAGGT				
MSP45	GGGAGCTCCTATGAATTACAGAGAATTGTTTAC	*A.ovis/A.marginale*	*MSP4*	851	de la Fuente et al., 2006 [56]
MSP43	CCGGATCCTTAGCTGAACAGGAATCTTGC				
groELf	TGAAGAGCATCAAACCCGAAG	*A. capra*	*Gro EL*	876	Yang et al., 2016 [57]
groELr	CTGCTCGTGATGCTATCGG

**Table 2:** PCR amplification conditions of *Anaplasma*

Category	Primer	Amplification conditions					
A. *bovis*	EE1, EE2	94°C	94℃	55°C	72°C	35	72°C
		5 min	30 s	30 s	30 s		5 min
	AB1f, AB1r	94°C	94°C	58°C	72°C	40	72°C
		5 min	30 s	30 s	30 s		10 min
A. *phagocytophilum*	EE1, EE2	94°C	94°C	55℃	72℃	35	72°C
		5 min	30 s	30 s	30 s		5 min
	SSAP2f, SSAP2r	94°C	94°C	58°C	72°C	40	72°C
		5 min	30 s	30 s	30 s		10 min
*A. ovis/ A. marginale*	MSP4f, MSP4r	94°C	94℃	54℃	72℃	35	72°C
		4 min	45 s	1 min	45 s		5 min
A. *capra*	groELf, groELr	94°C	94°C	55°C	72°C	35	72°C
		4 min	45 s	1 min	45 s		10 min

### Sequence variance comparison and sequence homology analysis

All *Anaplasma* positive PCR products were sequenced by Shanghai Bioengineering Sequencing Company. The sequences of the bidirectional sequencing results were spliced using DNAMAN 8.0 gene analysis software and logged into NCBI for sequence alignment. In this study, representative sequences were selected by multiple sequence alignment using the Clustal W program in MegAlign 7.2 (DNAStar, Madison, WI) software, and homology analysis of the sequences obtained in the study with known sequences was performed using this software. The selected sequences obtained in the study were analyzed by sequence difference comparison using DNAMAN 8.0 gene analysis software.

### Phylogenetic tree analysis

A phylogenetic tree was constructed using *16S rRNA*, *gro EL* (coding for a heat shock protein) and *msp4* (major surface protein 4) gene sequences. Strains from around the world were first obtained from GenBank and screened (i.e., duplicate sequenced, incomplete sequences were discarded and a sequence was selected in a subset of very similar sequences). Comparisons were performed by using the Clustal W in MegAlign software (DNAStar, Madison, WI). Then, the phylogenetic tree was constructed using Mega X software using the Kimura-2-parameter model in the Neighbor-joining (NJ) algorithm, and bootstrop values were obtained using 1000 replicates. Finally, the original tree exported by Mega X was stained and edited, and the software chosen for staining and editing was Figtree (http://tree.bio.ed.ac.uk/software/figtree/).

### Statistical analysis

Chi-square (χ2) and logistic regression tests (SPSS software, version 23) were used to assess the association between the molecular prevalence of *Anaplasma* and risk factors (age, gender, and feeding habits). Results were considered statistically significant for *P* < 0.05.

## Results

### Infection of *Anaplasma* spp. in cattle and goats in Hainan

An epidemiological study on *Anaplasma* infection in cattle and goats in Hainan province was carried out in Hainan Province from June 2018 to August 2020. A total of 731 goats from the 18 sampling regions in Hainan province were examined for the presence of *Anaplasma* spp.. Out of these, the samples were tested positive for *Anaplasma* spp. in 15 counties and negative in 3 counties. The infection rates were higher in Wenchang county (52%) and Chengmai county (76%) compared to other counties. The overall infection rate of *Anaplasma* spp. in goat samples was 30.1%. Categorically, the infection rates of *A. bovis*, *A. phagocytophilum*, *A. ovis* and *A. capra* were 22.7% (166), 13.8% (101), 5.2% (38) and 3.4% (25), respectively. *A. bovis* had the highest positive rate, followed by *A. phagocytophilum* and *A. ovis* (Table 3). In cattle samples, the infection rates of *A. bovis*, *A. phagocytophilum* and *A. marginale* were 11.4% (20), 6.3 (11), 5.7(10), respectively, with an overall infection rate of *Anaplasma* spp. at 14.8% (26) (Table 4). *A. bovis* had the highest positive rate, followed by *A. phagocytophilum*. *A. orvis* was only found in goats, and *A. marginale* was only found in cattle **(**Table 2). In both goat and cattle samples, the infection rates of *A.bovis* and *A. phagocytophilum* were relatively high, while *A. ovis* and *A. capra* were detected only in goat samples, and *A. marginale* was found in cattle. *A. capra*, a recently reported species of *Anaplasma*, is a new zoonotic pathogen that has been detected only in Chengmai county (Table 3).

**Table 3:** Anaplasma positivity rates in goats from Hainan, China

County	No. tested	No. infected/(%)			
		*A. ovis*	*A.phagocytophilum*	*A.bovis*	*A.capra*
Haikou	49	0(0/49)	0(0/49)	24.5(12/49)	0(0/49)
Qiongzhong	37	18.9(7/37)	2.7(1/37)	8.1(3/37)	0(0/37)
Dongfang	36	0(0/36)	2.7(1/36)	5.6(2/36)	0(0/36)
Wenchang	75	0(0/75)	44.0(33/75)	53.3(40/75)	0(0/75)
Changjing	32	18.8(6/32)	3.1(1/32)	0(0/32)	0(0/32)
Danzhou	29	20.7(6/29)	6.9 (2/29)	13.8(4/29)	0(0/29)
Dingan	65	0(0/65)	6.2 (5/65)	35.4(23/65)	0(0/65)
Wuzhishan	21	0(0/21)	0(0/21)	0(0/21)	0(0/21)
Qionghai	22	9.1(2/22)	4.5(1/22)	13.6 (3/22)	0(0/22)
Lingshui	26	1.0 (1/26)	0(0/26)	25.0 (6/26)	0(0/26)
Baisha	96	0(0/96)	29.2(28/96)	38.5(37/96)	0(0/96)
Chengmai	50	0(0/50)	54.0(27/50)	58.0(29/50)	50.0(25/50)
Baoting	21	0(0/21)	4.8(1/21)	4.8(1/21)	0(0/21)
Wanning	31	13.2(5/31)	6.4 (2/31)	16.1(5/31)	0(0/31)
Ledong	38	27.5(11/38)	0(0/38)	0(0/38)	0(0/38)
Tunchang	40	27.5(11/40)	0(0/40)	2.5(1/40)	0(0/40)
Lingao	34	0(0/34)	0(0/34)	0(0/34)	0(0/34)
Sanya	29	0(0/29)	0(0/290)	0(0/29)	0(0/29)
Total	731	5.2(38/731)	13.8(101/731)	22.7(166/731)	3.4(25/731)

**Table 4:** *Anaplasma* positivity rates in cattle from Hainan, China

County	No. tested	No. infected/(%)			
		*A. bovis*	*A. marginale*	*A. phagocytophilum*	*A. capra*
Dingan	161	6.8(11/161)	1.2(2/161)	1.2(2/161)	0(0/161)
Haikou	4	100.0(4/4)	25.0(1/4)	75.0(3/4)	0(0/4)
Qionghai	5	40.0(2/5)	80.0(4/5)	60.0(3/5)	0(0/5)
Danzhou	6	50.0(3/6)	50.0(3/6)	50.0(3/6)	0(0/6)
Total	176	11.4(20/176)	5.7(10/176)	6.3(11/176)	0(0/176)

### Mixed infection of *Anaplasma* spp.

The rate of infection with two or more *Anaplasma* spp. was 11.8% (86/731) in the731 goatsincluded in this study. Co-infection with *A. bovis* and *A. phagocytophilum* was 8.2% (60/731), followed by *A. bovis* + *A. ovis* (0.5%, 4/731), *A. bovis* + *A. capra* (0.4%, 3/731), *A. phagocytophilum* + *A.ovis* (0.1%, 3/731), and *A. bovis* + *A. capra* + *A. phagocytophilum* (2.0%, 15/731) (Table 5). In cattle, co-infections with two or three species of *Anaplasma* spp. were observed, with a positive co-infection rate of 6.8% (12/176) (Table 5). Specifically, the infection rate of *A. bovis* + *A. phagocytophilum* was 2.0% (3/176), and the infection rate of *A. bovis* + *A. phagocytophilum* was 1.1% (2/176). The infection rate of *A. phagocytophilum* + *A. marginale* was 0.6% (1/176), and the infection rate of *A. bovis* + *A. phagocytophilum* + *A. marginale* was 3.4% (6/176) (Table 5).

**Table 5:** Co-infection rates in goats and cattle samples

Identified Anaplasma species (Goat)	Positive(%)	Identified Anaplasma species (Bovine)	Positive(%)
*A.bovis* + *A.phago*	8.2(60/731)	*A.bovis* + *A.phago*	2.0(3/176)
*A.bovis* + *A.ovis*	0.5(4/731)	*A.bovis* + *A.marginale*	1.1(2/176)
*A.bovis* + *A.capra*	0.4(3/731)	*A.bovis* + *A.capra*	0(0/176)
*A.phago* + *A.capra*	0.4(3/731)	*A.phago* + *A.capra*	0(0/176)
*A.phago* + *A.ovis*	0.1(1/731)	*A.phago* + *A.marginale*	0.6(1/176)
*A.ovis* + *A.capra*	0(0/731)	*A.marginale* + *A.capra*	0(0/176)
*A.bovis* + *A.phago* + *A.ovis*	0(0/731)	*A.bovis* + *A.phago* + *A.marginale*	3.4(6/176)
*A.bovis* + *A.ovis* + *A.capra*	0(0/731)	*A.bovis* + *A.marginale* + *A.capra*	0(0/176)
*A.bovis* + *A.capra* + *A.phago*	2.0(15/731)	*A.bovis* + *A.marginale* + *A.capra*	0(0)
Total	11.8(86/731)		6.8(12/176)

### Prevalence distribution of Anaplasmosis by age, gender, and feeding mode in cattle and goats

According to the age, gender, and feeding habits, the infection of *Anaplasma* spp. in goats and cattle were investigated. A total of 37 male goats and 183 female goats were infected. The infection rate among male goats and female goats was 8.2% (37/122), 30.0% (183/609), respectively. There were differences in prevalence between the age groups and between the feeding habits. The infection rates were 20.0% (39/195), 30.2% (76/252), 36.8% (105/284) in goats younger than 1.5 years old, between 1.5 to 3 years old and older than 3 years old, respectively. Goats that were > 3 years old had the highest prevalence (36.8%) than those of other age groups (OR = 5.3, CI = 3.2–8.9, *P* < 0.001). The situation of *Anaplasma* spp. Infection differed between free-range and captive feeding modes of goats. The infection rate in free-range goats was 39.7% (115/390), significantly higher than that in captive goats (19.1%, 61/341) (OR = 4.3, CI = 3.0–6.3, *P* < 0.001).

For cattle, the infection rates in male and female were 14.3% (3/21) and 14.8% (23/155), respectively. The rates of *Anaplasma* spp. infections were 14.3% (9/63), 15.1% (13/86) and 11.1% (3/27) in cattle younger than 3 years, between 3 to 6 years, and older than 6 years, respectively. In the univariable statistical analysis, the feeding mode was associated with *Anaplasma* spp*.* infection, while no significant association was found between gender, age, and the prevalence of *Anaplasma* spp*.* (*P* > 0.05). Furthermore, the infection rate in free-range cattle was 73.7% (11/15), higher than that in the captive cattle (19.1%, 61/341) (OR = 28.4, CI = 7.7–104.9, *P* < 0.001) (Table 6).

**Table 6:** *Anaplasma* infection rates with respect to genders, ages, and feeding patterns

Animal	Parametrs	Categories	N	Positive	P%(SE)	Wald	OR(95% CI)	P-value
Goat	Gender	Female	609	37	8.2			
		Male	122	183	30.1(0.3)	1.8	0.7(0.4–1.2)	0.176
	Age group	< 1. 5 years	195	39	20.0	40.3	1	0.000
		1.5 to 3 years	252	76	30.2(0.2)	16.1	2.6(1.6–4.1)	0.000
		> 3 years	284	105	36.8(0.3)	40.2	5.3(3.2–8.9)	0.000
	Feeding system	Intensive rearing system	341	65	19.1			
		Free-range rearing system	390	155	39.7(0.2)	57.8	4.3(3.0–6.3)	0.000
Cattle	Gender	Female	155	23	14.8			
		Male	21	3	14.3			
	Age group	< 3 years	63	9	15.9			
		3 to 6 years	86	13	15.1			
		> 6 years	27	3	11.1			
	Feeding mode	Intensive rearing system	15	11	9.3		1	
		Free-range rearing system	161	15	73.3(0.7)	25.2	28.4(7.7–104.9)	0.000

### Sequence and phylogenetic analysis

#### Phylogenetic and sequence analysis of *A. phagocytophilum* based on 16S rRNA gene

In this study, 112 strains of *A. phagocytophilum 16S rRNA* sequences (641 bp) were obtained, of which 9 sequences contained polymorphic sites. Among them, 4 strains were derived from goats (CMAP30, BXAP37, HRAP1, ZQAP10), and 5 strains were derived from cattle (NDAAP2, RYAP3, RYAP4, RYAP6, XYAP2). The sequences of these 9 strains were compared with the reference strains (KJ782381), revealing 2 ~ 11 base site differences (Table 7).

**Table 7:** *16S rRNA* gene of *A.phagocytophilum* and their sequence base site differences

Number	Source	*Anaplasma phagocytophilum 16S rRNA* gene position							
		162	193	206	213	256	313	314	323
KJ782381	Sheep	C	G	T	G	A	A	T	T
NDaAP2	Cattle	*	A	*	A	*	*	-	C
CMAP30	Goat	T	*	*	A	*	T	*	C
BXAP37	Goat	*	A	*	A	*	*	-	C
RYAP3	Cattle	*	A	*	*	*	*	*	*
RYAP4	Cattle	*	A	*	*	*	*	*	*
RYAP6	Cattle	T	A	A	*	*	*	*	*
HRAP1	Goat	*	A	A	A	*	*	-	C
ZQAP10	Goat	*	A	*	A	G	*	-	C
XYAP2	Cattle	*	*	A	*	*	*	*	*
		461	589	591	598	599	603	614	616
KJ782381	Sheep	A	-	A	T	T	T	G	T
NDaAP2	Cattle	G	A	-	*	*	*	*	*
CMAP30	Goat	G	A	-	*	*	*	*	*
BXAP37	Goat	G	A	-	*	*	*	*	*
RYAP3	Cattle	*	*	*	*	*	*	A	G
RYAP4	Cattle	*	*	*	A	*	*	*	*
RYAP6	Cattle	*	*	*	*	*	*	A	G
HRAP1	Goat	G	A	-	*	*	*	*	G
ZQAP10	Goat	G	A	*	*	T	*	*	*
XYAP2	Cattle	*	*	*	*	*	C	A	C

^*^The same base

-The absence of base

**Fig. 2:**
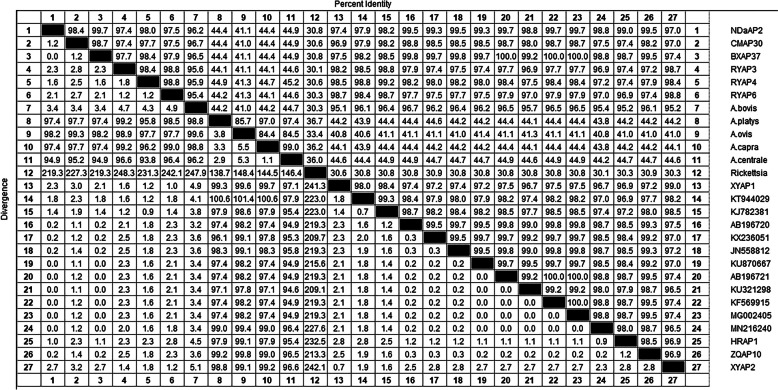
Sequences homology analysis of *A. phagocytophilum* based on *16S rRNA* gene

Homologous sequence analysis of *A. phagocytophilum* showed that the 9 *A. phagocytophilum 16S rRNA* sequences obtained in this study were compared with reference strains from China (KT944029, KJ782381, KU321298, KF569915, MG002405) and different countries’ reference strains, including South Africa (KU870667), Pakistan (MN216240), Japan (AB196720, AB196721), ranged from 97.4% to 100%. Meanwhile, the homology range with *A. bovis* (MH255938) was 95.6% ~ 98.8%, *A. ovis* (KJ459342) was 40.5% ~ 41.1%, and *Rickettsia* (JX885456) was 30.1% ~ 30.9%. *A. platys* (KU500907) has a homology range of 44.1% ~ 44.9%, and *A. capra* (MT052418) had a homology range of 44.1% ~ 44.7%. *A. centrale* (MH588233) had a homology range of 44.1% ~ 44.9% (Fig. 2).

**Fig. 3:**
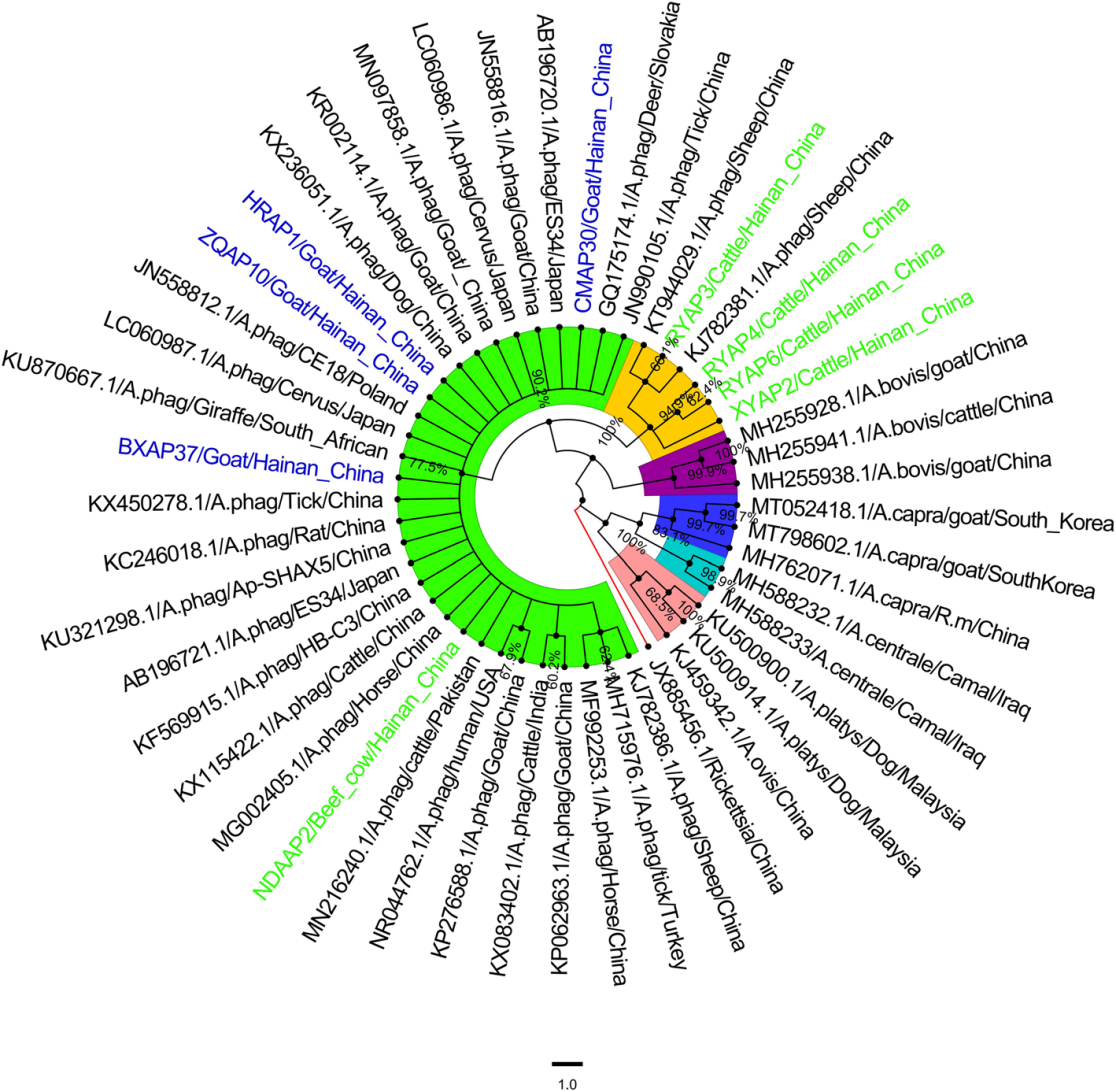
The phylogenetic analysis of the *A. phagocytophilum 16S rRNA* gene sequence (541 bp) by the neighbor-joining method. The number on each node corresponds to 1000 repeated guided analyses (only represents a percentage greater than 60%). The amplified sequences in this study are highlighted with colored fonts, different colored fonts indicate different animals (blue: goat;green: cow), and different branches are displayed with different colored backgrounds

The 9 *A. phagocytophilum 16S rRNA* sequences obtained in this study were compared with 27 known *A. phagocytophilum 16S rRNA* sequences from different regions (AB196720, AB19672, GQ175174, JN558812, JN558816, JN990105, KC246018, KF569915, KJ782381, KJ782386, KP062963, KP276588, KR002114, KT944029, KU321298, KU870667, KX083402, KX236051, KX236051, KX450278, LC060986, LC060987, MF992253, MG002405, MN097858, MN216240, NR044762), as well as *Rickettsia* (JX885456), *A. ovis* (KJ459342), *A. platys* (KU500900, KU500914, MH255941), *A. bovis* (MH255928, MH255938), *A.centrale* (MH588232, MH588233) and *A. capra* (MH762071, MT798602, MT052418) were used as outgroups to construct the phylogenetic tree. Phylogenetic tree analysis of *A. phagocytophilum* showed that the sequences from four goat strains (CMAP30, HRAP1, ZQAP10, BXAP37) and one bovine strain (NDAAP2) were grouped into one clade. The other clade from four bovine strains (XYAP2, RYAP3, RYAP4, and RYAP6) was isolated into a group. In this study, four types sequence derived from goat CMAP30, BXAP37, HRAP1 and ZQAP1 were compared with those from Japanese wild deer (AB196720), South African water giraffe (KU870667), Chinese dog (KX632051) and Zhejiang breed goat (JN558812), respectively. CMAP30 isolates had the longest genetic distance. In addition, the sequence of the bovine NDAAP2 strain in this study was similar to that of the bovine reference strain isolated from Pakistan (MN216240) (Fig. 3).

#### *A. bovis* sequence analysis and phylogenetic tree analysis

In this study, partial nucleotide sequences (551 bp) of *16S rRNA* of 168 strains *A. bovis* were obtained. All the obtained *A. bovis* sequences were compared, and 13 *A. bovis* strains were screened out as having sequences representing *16S rRNA.* Among them, 6 strains were derived from goats (CMAB16, YLAB7, CMAB24, LSAB11, HKAB10, BSAB89) and 7 strains were derived from cattle (RYAB3, RYAB6, RYAB7, RYAB9, XYAB2, DAAB145, DAAB155). The 13 *A. bovis* representative sequences were compared with the known reference strain (MN309843), and the results showed that there were differences in 1 to 4 base sites (Table 8).

**Table 8:** *16S rRNA* gene of *A.bovis* and the sequence base site differences

Number	Source	*Anaplasma bovis 16S rRNA* gene position												
		49	53	59	91	102	510	515	516	518	520	522	523	526
MN309843	Goat	A	A	A	A	-	T	T	T	G	G	T	G	T
CMAB16	Goat	*	*	*	G	T	*	*	*	*	*	*	*	*
YLAB7	Goat	*	*	-	*	T	*	*	*	*	*	*	*	*
CMAB24	Goat	*	*	-	G	T	*	*	*	*	*	*	*	*
DaAB145	Cattle	T	*	-	*	T	*	*	*	*	*	*	*	*
LSAB11	Goat	*	*	*	*	T	*	*	*	*	*	*	*	*
DaAB155	Cattle	*	*	*	*	A	*	*	*	*	T	*	*	*
HKAB10	Goat	*	-	-	*	T	*	*	*	*	*	*	*	*
BSAB89	Goat	*	*	-	G	T	*	*	*	*	*	*	*	*
RYAB3	Cattle	*	*	*	*	T	A	*	*	A	*	*	*	A
RYAB6	Cattle	*	*	-	*	T	*	*	*	*	*	G	*	*
RYAB7	Cattle	*	*	*	*	T	A	*	A	C	*	*	*	*
RYAB9	Cattle	*	*	*	*	T	*	A	*	A	*	G	*	*
XYAB2	Cattle	*	*	*	*	T	*	*	A	*	*	*	C	*

^*^The same base

-The absence of base

**Fig. 4:**
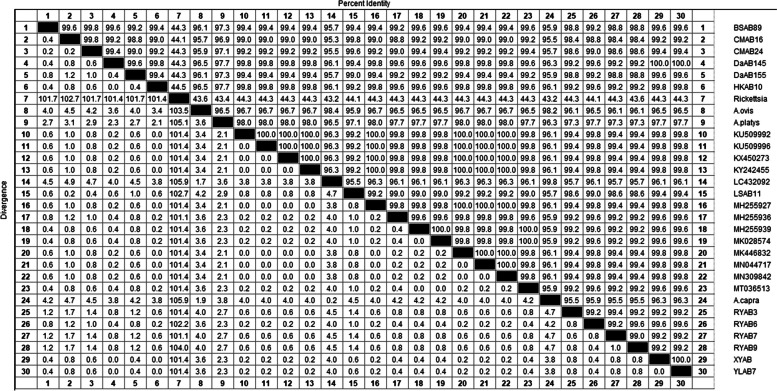
Sequences homology analysis of *A. bovis* based on *16S rRNA* gene

*A. bovis* homology analysis showed that the 13 *A. bovis 16S rRNA* sequences obtained in this study were compared with the *A. bovis* sequences from China and abroad (KU509992, KU509996, KX450273, KY242455,LC432092, MH255927, MH255936, MH255939, MN044717, MN309842, MT036513, MK028574) were analyzed with homology in the range of 95.5% ~ 99.8%. Meanwhile, the homology with *A. ovis* (KC484562) from Russia, *A. platys* (KU500900) from Malaysia, and *A. capra* (MT798602) from Korea were 95.7% ~ 96.9%, 96.9% ~ 99.8%, 95.5% ~ 96.3% respectively. The homology with *Rickettsia* (JX885456) from other area of China was 44.1% ~ 44.5% (Fig. 4).

**Fig. 5:**
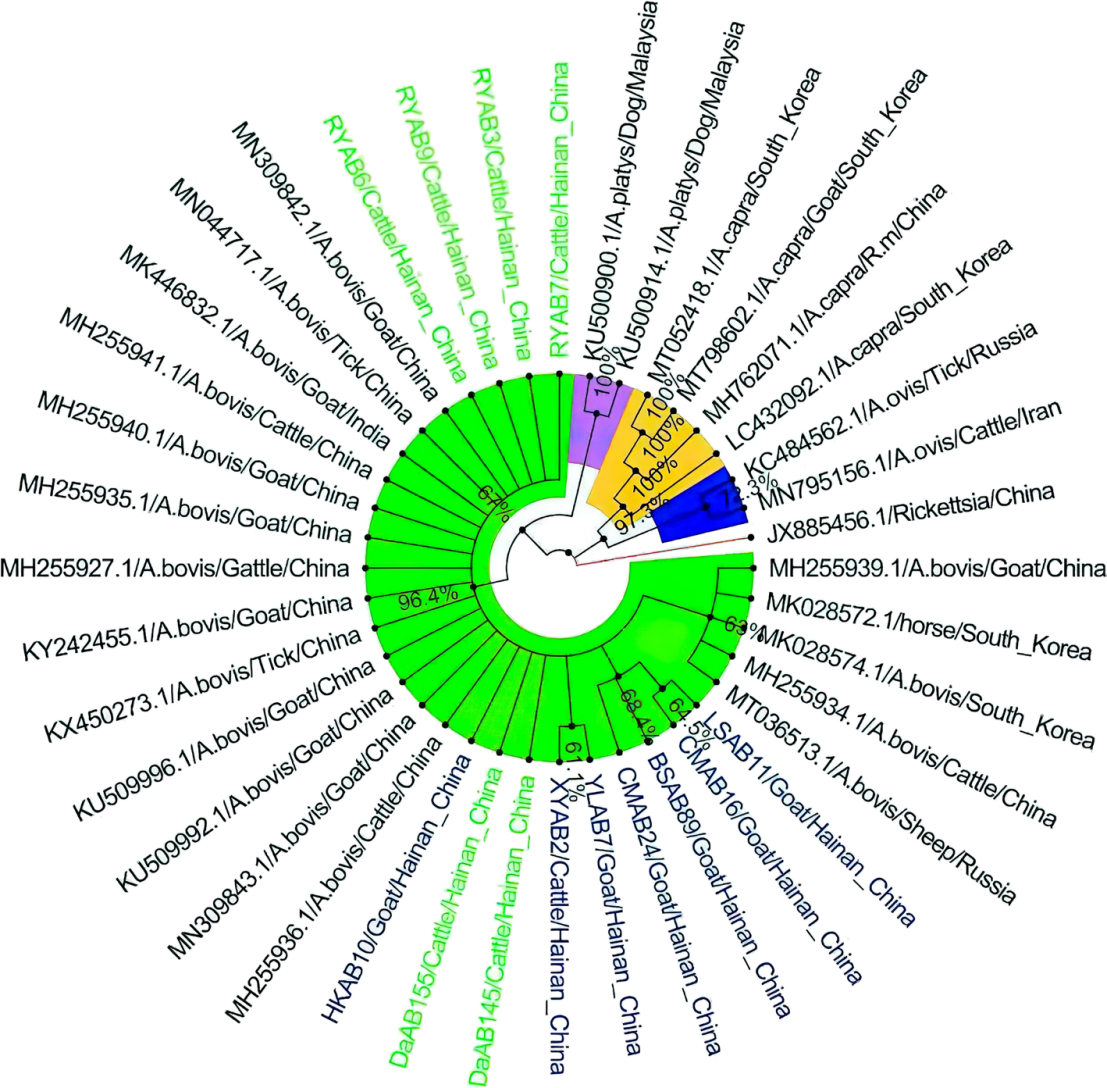
The phylogenetic analysis of the *16S rRNA* gene sequence (551 bp) of *A. bovis* by the neighbor joining method. The number on each node corresponds to 1000 repeated guided analyses (only represents a percentage greater than 60%).The amplified sequences in this study are highlighted with colored fonts, different colored fonts indicate different animals (blue: goat; green: cow), and different branches are displayed with different colored backgrounds. In addition, indicate the host or medium, country of origin, and GenBank accession number. Nine gene sequences of other strains as outgroups

In this study, 13 partial and 18 intact gene sequences of *A. bovis* 16S rRNA isolates from different regions were known (KU509992, KU509996, KX450273, KY242455, MH255927, MH255934, MH255935, MH255936, MH255939, MH255940, MH255941, MK028572, MK028574, MK446832, MN044717, MN309842, MN309843, MT036513). 16S rRNA gene sequences from *Rickettsia* (JX885456), *A. ovis* (KC484562, MH795156), *A. platys* (KU500900, KU500914) and *A. capra* (LC432092, MH762071, MT052418, MT798602) were used as outgroups to construct the phylogenetic tree. *A. bovis* phylogenetic tree analysis showed that *A. bovis* sequences from Hainan were clustered into a group with isolates from other provinces of China and other Asian countries (including Malaysia, Russia, and Korea), supported by 94% nodes (Fig. 5). In addition, 4 strains from goats (MAB16, YLAB7, CMAB24, LSAB11) and 2 strains from cattle (RYAB3, RYAB7) formed two separate subgroups on the same branch with 70% and 60% support on the branch nodes, respectively (Fig. 5).

#### Sequence and phylogenetic tree analysis of *A. capra*

**Fig. 6:**
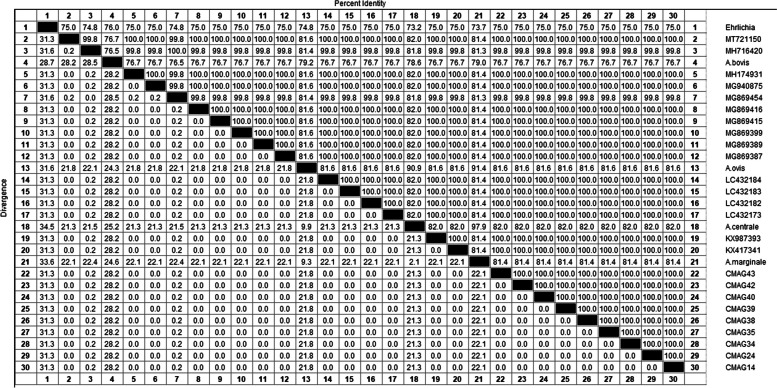
Sequences homology analysis of *A. capra* based on *Gro EL* gene

In this study, we obtained partial sequences of 25 strains *A. capra Gro EL* genes (878 bp), and found that they have only one sequence type through multiple sequence alignment. Homology analysis of *A. capra* showed that 9 strains (CMAG14, CMAG24, CMAG34, CMAG35, CMAG38, CMAG39, CMAG40, CMAG42, CMAG43) were selected and compared with those from other areas of China (MH716420, MH714931, MG940875, MG869454, MG869415, MG869416, MG869387, MG869388, MG869389, KX987394, KX417341) and from Korea (LC432173, LC432182, LC43218, LC4321824, MT721150) have a high homology of 99.8% to 100%. Meanwhile, the homology with other types of reference strains of *A. bovis* (MH255905), *A. ovis* (MG778623), *A. centrale* (KY522999), *A. marginale* (JQ839013), *Ehrlichia* sp. (U96731) was 76.7%, 81.6%, 82%, 81.4% and 75%, respectively (Fig. 6).

In this study, we obtained 9 strains *A. capra Gro EL* gene partial sequences and 17 strains *A. capra GroEL* gene reference sequences (LC432182, LC432184, MG869387, MG869389, MG869399, MG869415, MG869416, MG869454, MG940875, MH174931, MT721150, LC432183, LC432173, KX417341, KX417341, KX987393, MH716420) from different regions, and *GroEL* gene sequences of 2 strains *A. centrale* (KY522999, KY523000), 3 strains *A. marginale* (JQ839013, JQ839014, KY523034), 4 strains *A. ovis* (FJ460434, MG383905, MG778623), 2 strains *A. platys* (KU585953, KU585953) and 3 strains *A. bovis* (MH255905, MH255906, MH255907), were used as outgroups to construct phylogenetic trees.

**Fig. 7:**
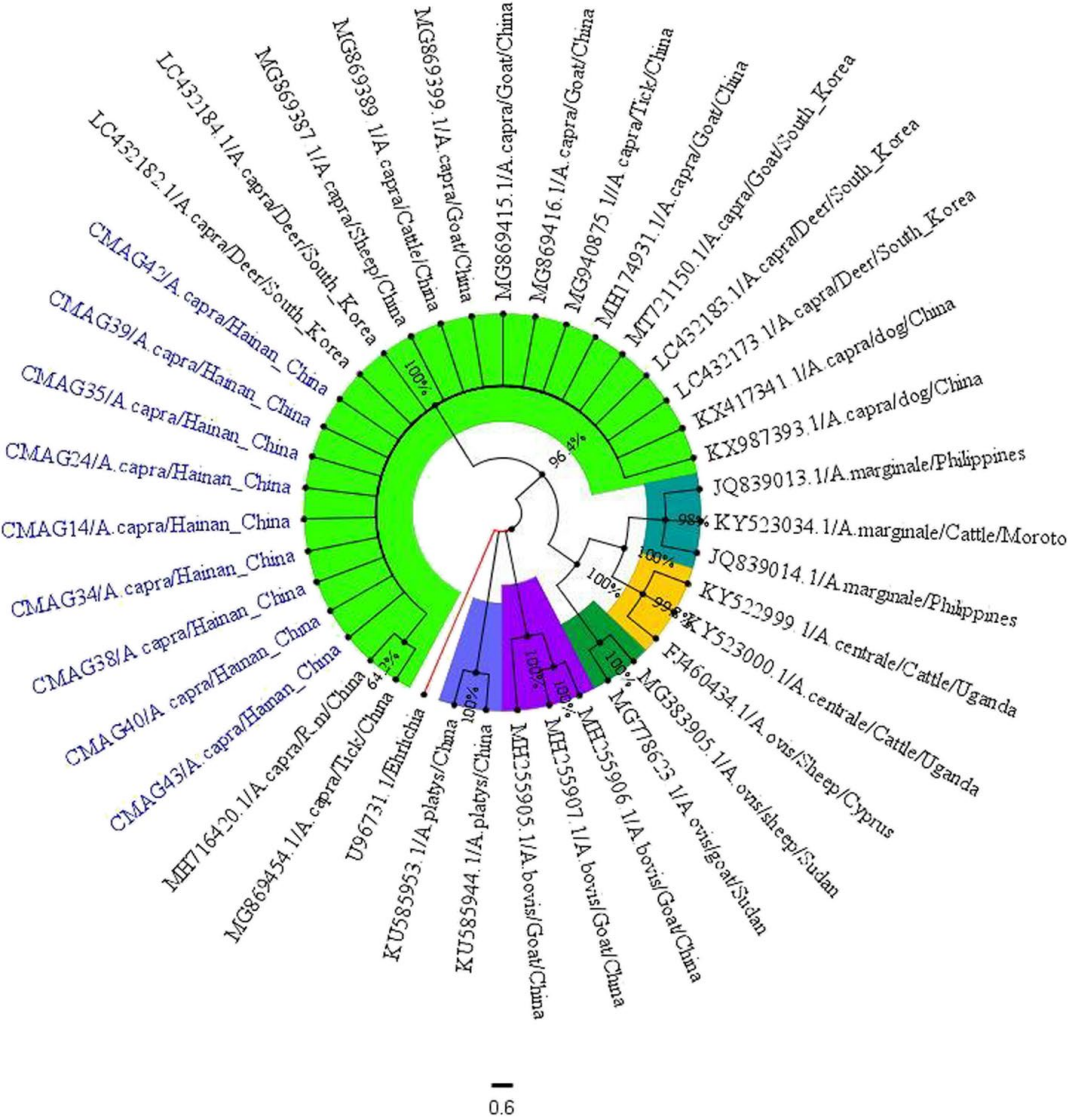
The phylogenetic analysis of the *A. capra Gro EL* gene sequence (878 bp) was performed using the neighbor-joining method. The number on each node corresponds to 1000 repeated guided analyses (only represents a percentage greater than 60%). The sequences amplified in this study are shown in red font. In addition, the GenBank accession number of he sequence used for phylogenetic analysis is also shown. Thirteen gene sequences of other strains as outgroups

The phylogenetic tree analysis of *A. capra* showed that 9 strains *A. capra* sequences and 17 strains reference strains all clustered in the same group in this study, and the support rate of branch nodes was as high as 100%. In addition, the genetic distance between CMAG43 sequence and other sequences was the longest (Fig. 7).

#### Sequence and phylogenetic tree analysis of *A. ovis*

In this study, 38 strains *A. ovis MSP4* partial gene sequences (851 bp) were obtained from goats, and 10 different sequences (TCAO11, TCAO40, QHAO19, SZAO26, LDAO19, EMAO14, DZAO1, CJAO4, CJAO16, BXAO11) were screened. The reference strain sequence (LC141091) from goats was compared with the selected representative sequences of 10 strains of *A. ovis*, and the results showed that there were differences in 3–20 base positions (Table 9).

**Table 9:** *MSP4* gene of *A. ovis* and the sequence base site differences

Numer	Source	*Anaplasma ovis msp4* gene position												
		50	55	58	64	68	72	79	87	89	92	104	288	388
LC141091	Goat	G	-	G	G	T	G	T	T	T	G	G	G	T
TCAO40	Goat	*	*	*	*	*	*	*	*	*	*	*	*	C
TCAO11	Goat	C	T	*	*	*	*	*	*	*	*	*	*	C
QHAO19	Goat	C	*	*	*	*	*	*	*	*	*	*	*	*
SZAO26	Goat	C	*	*	*	*	*	*	*	*	*	*	*	*
LDAO19	Goat	*	*	*	*	*	*	*	*	*	*	*	*	*
EMAO14	Goat	*	*	*	*	*	*	*	*	*	*	*	*	*
DZAO1	Goat	*	*	*	*	*	*	*	*	*	*	*	*	*
CJAO4	Goat	*	*	C	A	A	C	G	C	G	C	C	A	*
CJAO16	Goat	*	*	*	*	*	*	*	*	*	*	*	*	*
BXAO11	Goat	*	*	A	*	*	*	*	*	*	*	*	*	*
		461	490	493	516	688	778	803	813	817	820	829	840	846
LC141091		G	T	T	C	C	T	-	T	T	A	C	T	T
TCAO40	Goat	*	*	*	T	T	T	*	*	*	*	*	*	*
TCAO11	Goat	*	*	*	T	T	T	*	*	*	*	*	*	*
QHAO19	Goat	*	*	*	T	T	T	*	*	*	*	*	*	G
SZAO26	Goat	*	*	*	T	*	T	*	*	*	*	*	*	*
LDAO19	Goat	*	*	*	T	T	T	*	*	*	G	T	C	G
EMAO14	Goat	*	*	*	T	*	T	*	*	*	*	*	*	*
DZAO1	Goat	*	*	*	T	*	T	*	*	*	*	*	*	*
CJAO4	Goat	A	C	*	T	T	C	A	C	G	*	*	G	A
CJAO16	Goat	*	*	C	T	*	T	*	*	*	*	*	A	*
BXAO11	Goat	*	*	*	T	T	T	*	*	*	*	*	*	A

^*^The same base

-The absence of base

**Fig. 8:**
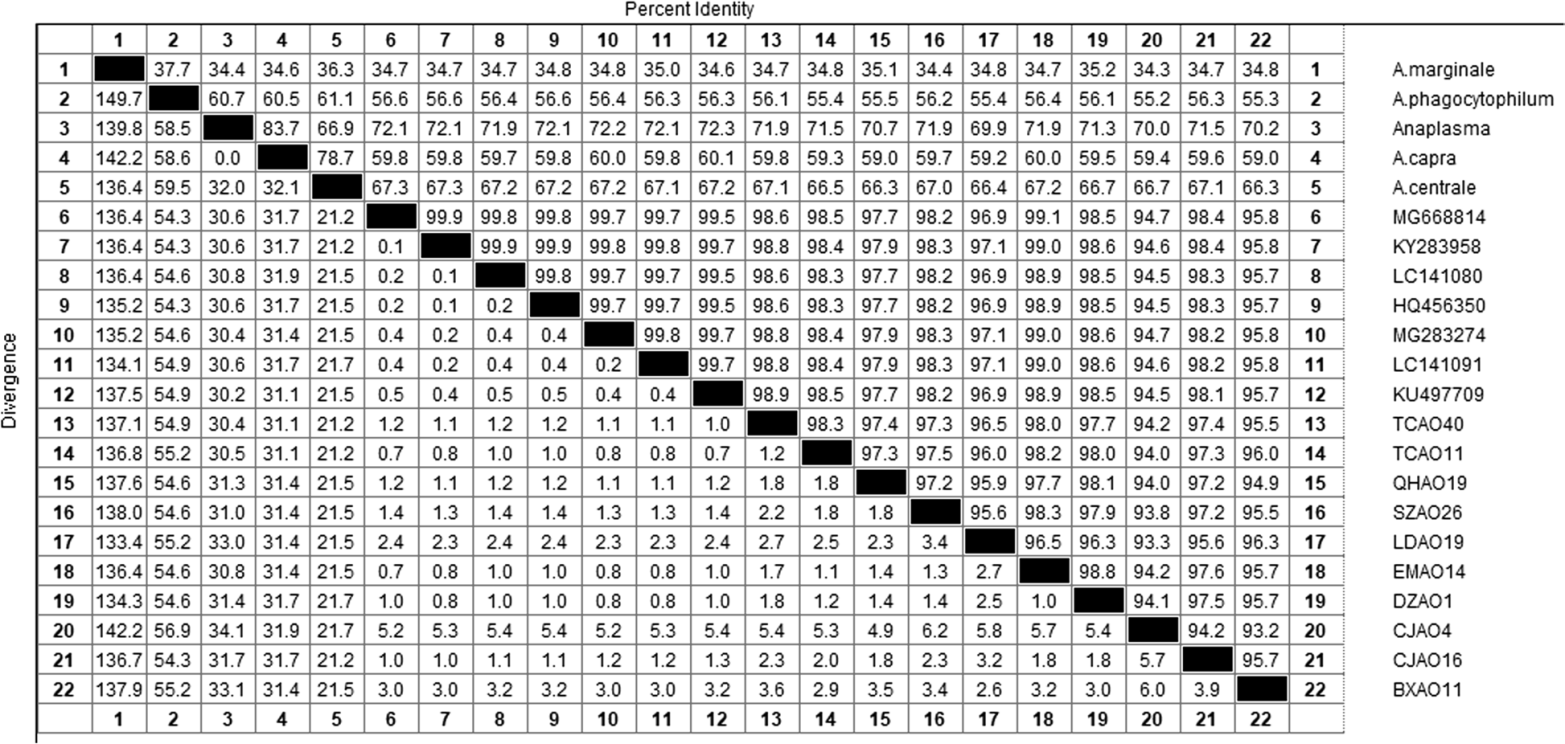
Sequences homology analysis of *A*. *ovis* based on *MSP4* gene

The homology analysis results of *A.ovis* showed that the partial gene sequences of 10 strains *A. ovis MSP4* obtained in this study were homologous to those of other Chinese strains (MG668814, HQ456350, MG283274), Turkish sheep (KY283958), Mongolian goats (LC141080, LC141091) and Sudan goats (KU497709), and the homology was 94.0% -99.0%. At the same time, the homology comparison with different species of *Anaplasma, A. marginale* (KX17990) from Algeria, *A. phagocytophilum (*KM205427) from Slovenia, *A. capra* (LC432231) from South Korea and *A. centrale* (KY305621) from South Africa were 34.4%—35.2%, 55.2%—56.4%, 66.3%—67.2% and 59.0%—60.0%, respectively (Fig. 8).

In this study, we obtained 10 strains *A. ovis MSP4* gene partial sequences, and 18 strains *A. ovis MSP4* gene reference sequences (GQ925816, HQ456350, KC432643, KU497709, KU497710, KU497712, KY283958, LC141077, LC141080, LC141081, LC141091, MG283274, KP608305, MG668814, MG564176, MN198191, MK358053, MH790274) from different regions, and *MSP4* gene sequences of 3 strains *A. centrale (*KY305601, KY305620, KY305621), 2 strains *A. marginale* (KX179906, KX179906), 2 strains *A. capra* (MK83607, LC432231) and 1 strains *A. phagocytophilum* (EU008082), were used as outgroups to construct phylogenetic trees. The phylogenetic tree analysis of *A. ovis* showed that *A. ovis, A. centrale*, *A. marginale*, *A. capra* and *A. phagocytophilum* independently clustered into different clades.

**Fig. 9:**
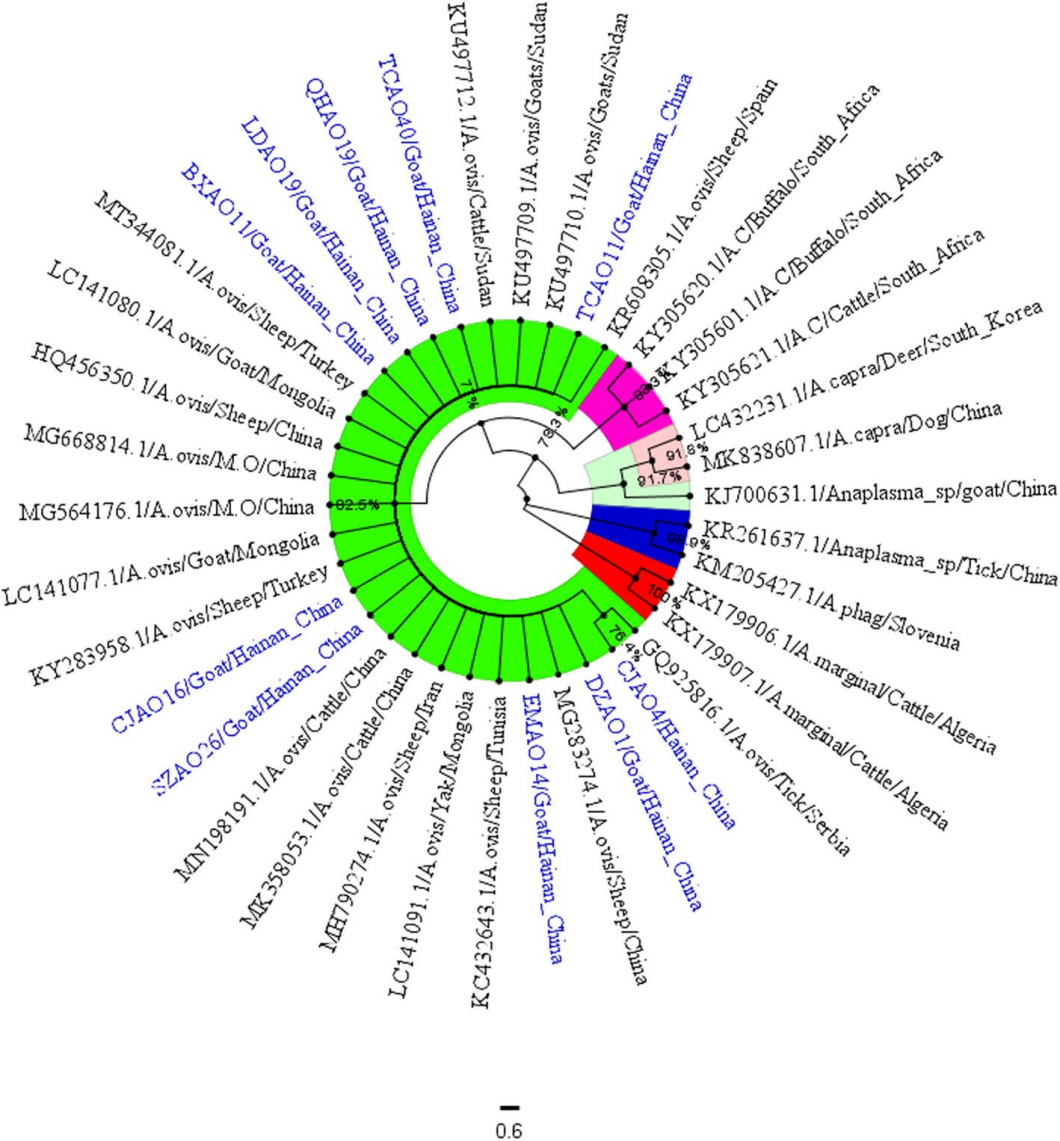
The phylogenetic analysis of *A. ovis MSP4* gene sequence (851 bp) by the neighbor joining method. The number on each node corresponds to 1000 repeated guided analyses (only represents a percentage greater than 60%). The amplified sequences in this study are shown in blue font. In addition, the GenBank accession number of the sequence used for phylogenetic analysis is also shown. 11 gene sequences of other strains as outgroups

In this study, 10 *A. ovis* sequences and 18 strains known reference sequences were clustered into the same group, and the branch node support rate was 82.5%. However, the genetic distance between CJAO_4_ and other sequences is far (Fig. 9).

#### Analysis of *A. marginale* sequence and phylogenetic tree

Ten *A. marginale MSP4* gene sequences (DAAM9, DAAM95, RYAM2, RYAM3, RYAM 4, RYAM5, RYAM6, RYAM7, RYAM9, XYAM4) were obtained in this study. The sequences of the 10 *A. marginale MSP4* strains obtained in this study were compared with the known reference strain (MK809386), and the results showed that there were differences at 3 ~ 9 base positions. The results showed that there were differences in 3 ~ 9 base positions (Table 10).

**Table 10:** *MSP4* of *A. marginale* and the sequencebase site differences

Number	Source	*Anaplasma marginale msp4* gene position												
		21	23	39	43	46	48	52	57	60	74	91	92	93
MK809386		A	T	C	G	C	T	G	-	G	-	-	G	T
DAAM9	Cattle	*	*	*	C	*	C	*	*	C	*	G	T	G
DAAM95	Cattle	*	*	*	*	*	*	T	*	*	*	*	*	*
RYAM2	Cattle	*	*	*	*	A	*	*	*	*	G	*	*	*
RYAM3	Cattle	*	*	A	C	A	*	*	T	*	*	*	*	*
RYAM4	Cattle	*	*	*	*	*	*	T	*	*	*	A	*	*
RYAM5	Cattle	*	-	*	*	*	-	*	*	*	*	*	*	*
RYAM6	Cattle	*	*	*	*	*	*	T	*	*	*	*	*	*
RYAM7	Cattle	*	*	*	*	A	*	C	T	*	*	*	*	*
RYAM9	Cattle	T	M	A	*	*	G	*	*	*	*	*	*	*
XYAM4	Cattle	*	*	*	C	A	*	*	*	*	*	*	*	*
		323	581	715	768	780	805	814	826	828	833	834	835	840
MK809386		C	C	-	-	-	-	-	G	-	-	T	G	-
DAAM9	Cattle	*	A	*	*	*	*	*	*	T	*	*	*	T
DAAM95	Cattle	*	*	*	*	*	A	T	*	*	T	*	*	C
RYAM2	Cattle	*	*	*	*	*	A	*	*	*	T	-	*	C
RYAM3	Cattle	*	*	*	*	*	*	*	*	*	A	*	*	C
RYAM4	Cattle	*	*	*	*	*	*	C	A	*	T	-	*	T
RYAM5	Cattle	*	*	*	*	*	*	*	*	*	*	*	*	*
RYAM6	Cattle	T	*	*	*	*	*	*	*	*	*	*	*	*
RYAM7	Cattle	*	*	*	*	*	*	*	*	*	*	*	*	*
RYAM9	Cattle	*	*	*	*	*	*	*	*	*	*	G	C	*
XYAM4	Cattle	*	*	T	*	*	*	*	*	*	*	*	*	*

^*^The same base

-The absence of base

**Fig. 10:**
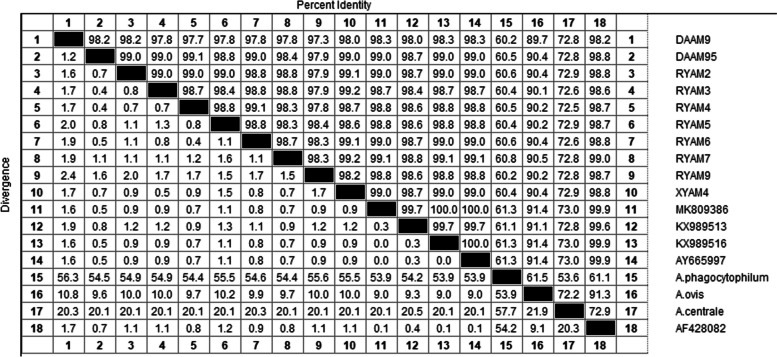
Sequences homology analysis of *A. marginale* based on *MSP4* gene

In the present study, 10 *A. marginale MSP4* partial gene sequences were analyzed for homology with reported domestic and international sequences (MK809386, KX989513, KX989516, AY665997, AF428082), and their homology ranged from 98.3.0% to 99.1%. The homology analysis with *A. phagocytophilum* (EU180058), *A. centrale* (KY305621) and *A. ovis* (KU499307) was also performed, and the homology ranged from 62.5% ~ 62.8%, 72.5% ~ 72.9%, and 89.7% ~ 90.5%, respectively (Fig. 10).

**Fig. 11:**
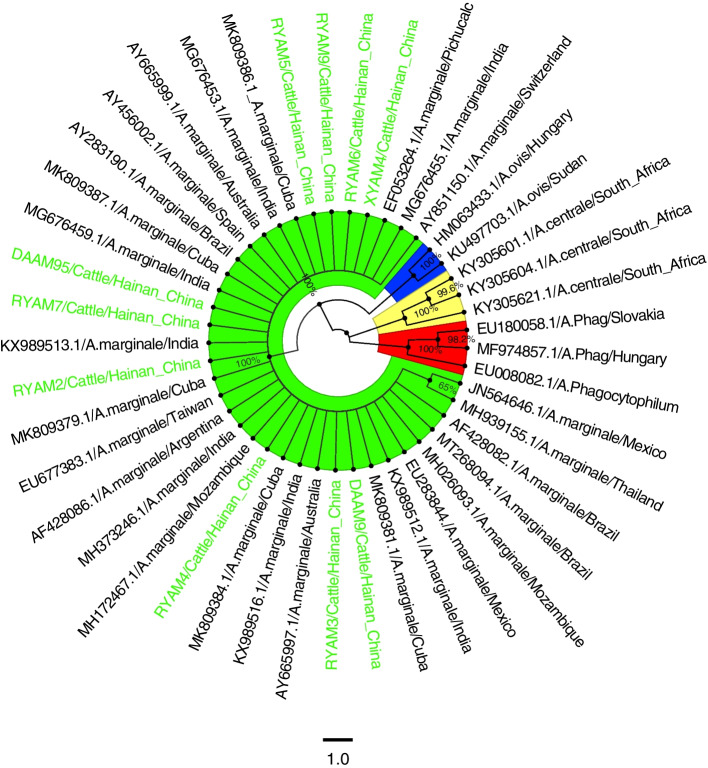
The phylogenetic analysis of *A. marginale MSP4* gene sequence (851 bp) by the neighbor joining method. The number on each node corresponds to 1000 repeated guided analyses (only represents a percentage greater than 60%). The amplified sequences in this study are shown in blue font. In addition, the GenBank accession number of the sequence used for 11 gene sequences of other strains as outgroup

The partial sequences of the 10 *A. marginale MSP4* genes obtained in this study were compared with the known sequences of 27 *A. marginale MSP4* genes from different regions (AF428082, AF428086, AY283190, AY456002, AY665997, AY665999, AY851150, EF053264, EU283844, EU677383, JN564646, KX989516, KX989512, KX989513, MG676453, MG676455, MG676459, MH026093, MH172467, MH373246. MH939155, MK809379, MK809381, MK809384, MK809386, MK809387, MT268094), two strains of *A. ovis* (HM063433, KU497703), three strains of *A. centrale* (KY305601, KY305604, KY305621) and three strains of *A. phagocytophilum* (EU008082, EU180058, MF974857) were used as outgroups for *MSP4* gene sequences to construct phylogenetic trees. Phylogenetic tree analysis based on the *MSP4* gene showed that the *MSP4* gene sequences of *A. marginale, A. phagocytophilum, A. centrale* and *A. ovis* were independent and clustered into different branches. The sequences of 10 *A. marginale* strains obtained in this study clustered into one taxon with the known reference sequences of 27 *A. marginale* strains, with 100% support of branching nodes, among which DAAM9 strains were genetically distant (Fig. 11).

## Discussion

It has been reported that *Anaplasma* spp. are widely distributed in China, and their DNA has been found in a variety of wild and domestic animals and ticks [37, 50, 59–62]. This study was conducted for molecular detection and phylogenetic analysis of *Anaplasma* pathogens based on *16S rRNA*, *Gro EL* and *MSP4* as marker genes in ruminants from Hainan province/island. This study reported the prevalence of anaplasmosis in ruminants in Hainan province/island.

The overall prevalence of anaplasmosis in cattle and goats in Hainan province was recorded as 27.1% (246/907), which was lower than the prevalence reported in small ruminants in Anhui province in central China 33.0% (67/203). The prevalence in goats and cattle was 30.1% (220/731) and 14.8% (26/176), respectively, which were higher than the prevalence reported in cows in Xinjiang in the north west of China (3.2%, 16/493), but lower than those reported in sheep in Heilongjiang in the north of China (30.2%, 103/341). The prevalence of anaplasmosis in ruminant was also reported in other Asian countries, including cattle in Iran, 15.5% [63], cattle in Malaysia, 60.7% [64], cattle in Mozambique, 86.3% [65], Cattle in Algeria, 42.2% [66], sheep, 35.6% and goats, 46% in northern Tunisia [67]. These differences may be due to different geographical areas, climatic conditions [68], different diagnostic methods used, seasons of sample collection, tick infestation intensity, and animal breeds with different susceptibility to pathogens.

In this study, *A. bovis* was more prevalent than other *Anaplasma* spp. which is consistent with previous studies from different parts of China. Previous studies have shown that the prevalence of *A. bovis* varied in other areas of China, ranging from 4.5% to 49.0% [58, 61, 69–72]. The feeding method was also significantly associated with the prevalences. In this study, the prevalence in ruminants in free-range farming was higher than that in intensive rearing system. This result was consistent with another study that reported animals browsing outdoor were at higher risk than the animals fed indoor [73]. It may be due to conditions controlled in large-scale farming environments that reduce the incidence of tick bites. Under free range conditions, there is an increased incidence of tick bites, which may lead to a higher rate of *Anaplasma* spp. infection. The prevalence of *Anaplasma* spp. infection was higher in old animals, > 3 years (36.8%, 105/204) than in younger animals, < 1.5 years of age (20%, 39/195) and those between 1.5 and 3 years of age (30.2%, 76/252). The difference in the prevalence in goats at different ages was possibly because older goats had a higher chance of being bitten by ticks.

*A. phagocytophilum* has been recognized as a zoonotic pathogen. In 1994, *A. phagocytophilum* was first identified as the pathogen of HGA in the United States. In China, the first suspected human case was reported in Anhui province in 2006. Recently, *A. capra*, a newly reported species originally found in goats; caused 28 infections in people reported in Heilongjiang province in China in 2015 [1, 36]. An *Anaplasma* sp. was first detected in a Cyprus patient with fever, hepatosplenomegaly, and lymph node disease in 2006 [74]. In addition, two cases of flat anplasmosis were detected in two women in Venezuela [45]. To date, besides *A. phagocytophilum* infections in humans, there have been relatively few reports of *A. capra*, *A. ovi*s and *A. platys* human infections. However, they are all potential zoonotic pathogens, which are easily overlooked in disease prevention [75]. The study aimed to investigate the presence, prevalence, and genotypes of *A*. *phagocytophilum*, *A*. *ovis*, and *A*. *capra* in sheep from Kyrgyzstan. Polymerase chain reaction (PCR), restriction fragment length polymorphism (RFLP), and sequencing techniques targeting the 16S SSU rRNA, groEL, and gtlA genes were employed. The findings revealed, for the first time, the presence of A. phagocytophilum-like 1, *A*. *ovis*, and *A*. *capra* in sheep from Kyrgyzstan. The positivity rates for A. phagocytophilum-like 1, *A*. *ovis*, and *A*. *capra* genotype-1 were 6.9%, 22.5%, and 5.3%, respectively. It was also observed that *A*. *capra* exhibits two distinct genotypes, namely *A*. *capra* genotype-1 and *A*. *capra* genotype-2 [76].

In the past, anaplasmosis in ruminants was a blood borne disease that was easily overlooked, but in recent years, with the widespread increase in incidence trends, it will be increasingly valued. Clinical symptoms of cattle and sheep infected with Anaplasma include anemia, emaciation, jaundice, and other clinical symptoms, sometimes leading to the death of cattle and sheep, causing serious economic losses in pastoral areas.

A. Phagocytophilum is a zoonotic pathogen, and HGA poses a serious threat to human health. This study aims to investigate the 16S rRNA/Gro EL/MSP4 gene of this pathogen, which can help to trace its transmission and understand whether there are host differentiation and geographical differentiation characteristics of the Anaplasma pathogen in Hainan region, thus providing favorable reference materials for the prevention and control of this disease.

## Conclusion

This study investigated the molecular epidemiology of anaplasmosis in ruminants in Hainan province/island, China. The total infection rate in goats was 30.1%. The infection rates of *A. bovis*, *A. phagocytophilum*, *A. ovis* and *A. capra* were 22.7%, 13.8%, 5.2% and 3.4%, respectively. The infection rate in goats with two or more species of *Anaplasma* was 11.8%. The rate of *Anaplasma* infection in cattle was 14.8%. The infection rates of *A. bovis*, *A. phagocytophilum* and *A. marginale* in cattle were 11.4%, 6.3% and 5.7%, respectively. The co-infection rate of *Anaplasma* in cattle was 11.8%. The results showed that *Anaplasma* was prevalent in ruminants in Hainan province, China and co-infection was common. In this study, 1*6S rRNA/ Gro EL/MSP4* gene phylogenetic tree analysis showed that *A. phagocytophilum, A. bovis*, *A. ovis*, *A. capra*, and *A. marginale* did not exhibit characteristics of geographical isolation and differentiation from isolates in different regions in Hainan. However, the phylogenetic tree of *A. phagocytophilum* revealed distinct branches associated with different hosts, suggesting host differentiation. Hainan province is located in tropical and subtropical regions, which are more suitable for the growth and reproduction of certain tick species. However, the specific tick species carrying *Anaplasma* spp*.* in Hainan are unknown. This study conducted a molecular epidemiological investigation on *Anaplasma* spp*.* in ruminants in Hainan, providing a favorable basis for understanding the spread, prevention, and treatment of these infections in the province*.*

In conclusion, anaplasmosis represents a potential threat to the ruminant husbandry in Hainan, and our studies have also shown that co-infection of *Anaplasma* spp. is common. The present study demonstrated that a significant proportion of cattle and goats infected with *Anaplasma* spp., even though none of the animals showed clinical symptoms. These animals act as carriers of the bacteria. It is important for farmers, local veterinarians, and the local government in Hainan to take preventive measures against anaplasmosis in domestic animals.

## Data Availability

The data that support the findings of this study are available from the corresponding author upon reasonable request. Extracted DNA of blood samples will be made available upon request in case there is leftover material.

## References

[1] Li H, Zheng YC, Ma L (2015). Human infection with a novel tick-borne Anaplasma species in China: a surveillance study. Lancet Infect Dis.

[2] Ismail N, McBride JW (2017). Tick-Borne Emerging Infections: Ehrlichiosis and Anaplasmosis. Clin Lab Med.

[3] Henningsson Anna J (2015). Low risk of seroconversion or clinical disease in humans after a bite by an Anaplasma phagocytophilum-infected tick. Ticks Tick Borne Dis.

[4] Alberti A, Zobba R, Chessa B (2005). Equine and canine Anaplasma phagocytophilum strains isolated on the island of Sardinia (Italy) are phylogenetically related to pathogenic strains from the United States. Appl Environ Microbiol.

[5] Ben Said M, Belkahia H, El Mabrouk N (2017). Molecular typing and diagnosis of Anaplasma spp. closely related to Anaplasma phagocytophilum in ruminants from Tunisia. Ticks Tick Borne Dis.

[6] Said MB, Belkahia H, Messadi L (2018). Anaplasma spp. in North Africa: A review on molecular epidemiology, associated risk factors and genetic characteristics. Ticks Tick Borne Dis.

[7] Duscher GG, Battisti E, Hodžić A (2020). First detection and molecular identification of Anaplasma phagocytophilum in an introduced population of Reeve's muntjac (Muntiacus reevesi) in United Kingdom. Mol Cell Probes.

[8] Lappin MR (2018). Update on flea and tick associated diseases of cats. Vet Parasitol.

[9] Stuen S, Granquist EG, Silaghi C (2013). Anaplasma phagocytophilum–a widespread multi-host pathogen with highly adaptive strategies. Front Cell Infect Microbiol.

[10] Selmi R, Ben Said M, Dhibi M (2020). Genetic diversity of gro EL and msp4 sequences of Anaplasma ovis infecting camels from Tunisia. Parasitol Int.

[11] Stuen S, Longbottom D (2011). Treatment and control of chlamydial and rickettsial infections in sheep and goats. Vet Clin North Am Food Anim Pract.

[12] Renneker S (2013). Can Anaplasma ovis in Small Ruminants be Neglected any Longer?. Transbound Emerg Dis.

[13] Kocan KM, de la Fuente J, Blouin EF (2010). The natural history of Anaplasma marginale. Vet Parasitol.

[14] Asif M, Parveen A, Ashraf S, et al. First Report Regarding the Simultaneous Molecular Detection of Anaplasma marginale and Theileria annulata in Equine Blood Samples Collected from Southern Punjab in Pakistan[J]. Acta Parasitologica. 2020;65(1):259–63. 10.2478/s11686-019-00141-w 31721059

[15] Guillemi EC, Imbert M, de la Fournière S (2020). Closing the gaps to understand the tick transmission of Anaplasma marginale among Giant Anteaters (Myrmecophaga tridactyla) in Argentina. Pathogens.

[16] Hernández-Velasco A, Sánchez-Montes S, Romero-Salas D (2020). First record of natural infection with Anaplasma marginale in sucking lice infesting the water buffalo (Bubalus bubalis) in Mexico. Parasitol Res.

[17] Martínez-Ocampo F, Quiroz-Castañeda RE, Amaro-Estrada I (2020). Whole-Genome Sequencing of Mexican Strains of Anaplasma marginale: An Approach to the Causal Agent of Bovine Anaplasmosis. Int J Genomics.

[18] Zeb J, Shams S, Din IU (2020). Molecular epidemiology and associated risk factors of Anaplasma marginale and Theileria annulata in cattle from North-western Pakistan. Vet Parasitol.

[19] Díaz-Sánchez AA, Meli ML, Álvarez DO (2020). Development and application of a multiplex TaqMan® real-time qPCR assay for the simultaneous detection of Anaplasma marginale and Theileria annulata and molecular characterization of Anaplasma marginale from cattle in Western Cuba[J]. Ticks Tick Borne Dis.

[20] Guarnizo TRM, Alvarez DO, Díaz-Sánchez AA (2020). Epidemiology and genetic diversity of Anaplasma marginale in Zamora-Chinchipe, Ecuador. Ticks Tick Borne Dis.

[21] Said MB, Asker AB, Belkahia H (2018). Genetic characterization of Anaplasma marginale strains from Tunisia using single and multiple gene typing reveals novel variants with an extensive genetic diversity. Ticks Tick Borne Dis.

[22] Byaruhanga C, Nicola EC, Darryn LK (2018). Molecular detection and phylogenetic analysis of Anaplasma marginale and Anaplasma centrale amongst transhumant cattle in north-eastern Uganda. Ticks Tick Borne Dis.

[23] Mutshembele AM, Cabezas-Cruz A, Moses S (2014). Epidemiology and evolution of the genetic variability of Anaplasma marginale in South Africa. Ticks Tick Borne Dis.

[24] Nair AS, Ravindran R, Lakshmanan B (2013). Bovine carriers of Anaplasma marginale and Anaplasma bovis in South India. Trop Biomed.

[25] Ashraf S, Parveen A, Awais MM, et al. A Report on Molecular Detection and Phylogenetic Evaluation of Anaplasma marginale in Ticks and Blood Samples Collected from Cattle in District Layyah in Punjab (Pakistan). Curr Microbiol. 2021;78(1):274–81. 10.1007/s00284-020-02256-0. 10.1007/s00284-020-02256-0 33125524

[26] Junsiri W, Watthanadirek A, Poolsawat N (2020). Molecular detection and genetic diversity of Anaplasma marginale based on the major surface protein genes in Thailand. Acta Tropica.

[27] Nguyen AHL, Tiawsirisup S, Kaewthamasorn M (2020). Molecular detection and genetic characterization of Anaplasma marginale and Anaplasma platys-like (Rickettsiales: Anaplasmataceae) in water buffalo from eight provinces of Thailand. BMC Vet Res.

[28] Atambekova Zhyldyz, Thillaiampalam Sivakumar, Ikuo Igarashi (2019). Epidemiological survey of Anaplasma marginale in cattle and buffalo in Sri Lanka. J Vet Med Sci.

[29] Jurković D, Mihaljević Ž, Duvnjak S (2020). First reports of indigenous lethal infection with Anaplasma marginale, *Anaplasma bovis* and *Theileria orientalis* in Croatian cattle. Ticks Tick Borne Dis.

[30] Atif FA (2016). Alpha proteobacteria of genus Anaplasma (Rickettsiales: Anaplasmataceae): Epidemiology and characteristics of Anaplasma species related to veterinary and public health importance. Parasitology.

[31] Rar V, Golovljova I (2011). Anaplasma, Ehrlichia, and "Candidatus Neoehrlichia" bacteria: pathogenicity, biodiversity, and molecular genetic characteristics, a review. Infect Genet Evol.

[32] Ben Said M, Belkahia H, Karaoud M (2015). First molecular survey of Anaplasma bovis in small ruminants from Tunisia. Vet Microbiol.

[33] Goethert HK, Telford SR (2003). Enzootic transmission of Anaplasma bovis in Nantucket cottontail rabbits. J Clin Microbiol.

[34] Sasaki H (2012). Molecular survey of Rickettsia, Ehrlichia, and Anaplasma infection of domestic cats in Japan[J]. Ticks Tick Borne Dis.

[35] Seo MG, Kwon OD, Kwak D (2019). Anaplasma bovis infection in a horse: First clinical report and molecular analysis. Vet Microbiol.

[36] Beyer AR, Jason AC (2015). Of goats and men: rethinking anaplasmoses as zoonotic infections. Lancet Infect Dis.

[37] Shi Y, Yang J, Guan G (2020). Molecular investigation of Anaplasma species in sheep from Heilongjiang Province, northeast China identified four Anaplasma species and a novel genotype of Anaplasma capra. Parasitol Int.

[38] Peng Y, Wang K, Zhao S (2018). Detection and Phylogenetic Characterization of Anaplasma capra: An Emerging Pathogen in Sheep and Goats in China. Front Cell Infect Microbiol.

[39] Altay K, Erol U, Sahin OF (2022). The first molecular detection of Anaplasma capra in domestic ruminants in the central part of Turkey, with genetic diversity and genotyping of Anaplasma capra. Trop Anim Health Prod.

[40] Seo MG, Ouh IO, Lee H (2018). Differential identification of Anaplasma in cattle and potential of cattle to serve as reservoirs of Anaplasma capra, an emerging tick-borne zoonotic pathogen. Vet Microbiol.

[41] Amer S, Kim S, Yun YM (2019). Novel variants of the newly emerged Anaplasma capra from Korean water deer (Hydropotes inermis argyropus) in South Korea. BioMed Central.

[42] Jouglin M, Blanc B, de la Cotte N (2019). First detection and molecular identification of the zoonotic Anaplasma capra in deer in France. PLoS One.

[43] Sahin OF, Erol U, Altay K (2022). Buffaloes as new hosts for Anaplasma capra: Molecular prevalence and phylogeny based on gtlA, groEL, and 16S rRNA genes. Res Vet Sci.

[44] Altay K, Erol U, Sahin OF (2022). First molecular detection of Anaplasma species in cattle from Kyrgyzstan; molecular identification of human pathogenic novel genotype Anaplasma capra and Anaplasma phagocytophilum related strain. Ticks Tick Borne Dis.

[45] Arraga-Alvarado CM, Qurollo BA, Parra OC (2014). Case report: Molecular evidence of Anaplasma platys infection in two women from Venezuela. Am J Trop Med Hyg.

[46] Gaowa W, Yin XH, et al. Case of Human Infection with Anaplasma phagocytophilum in Inner Mongolia, China[J]. Jpn J Infect Dis. 2018;71(2):155–7. 10.7883/yoken.JJID.2017.450. 10.7883/yoken.JJID.2017.450 29491236

[47] Guo WP, Zhang B, Wang YH (2019). Molecular identification and characterization of Anaplasma capra and Anaplasma platys-like in Rhipicephalus microplus in Ankang, Northwest China[J]. BMC Infect Dis.

[48] Qin XR, Han FJ, Luo LM (2018). Anaplasma species detected in Haemaphysalis longicornis tick from China[J]. Ticks Tick Borne Dis.

[49] Xi-Feng Sun (2015). Anaplasma species in China. Lancet Infect Dis.

[50] Yan Y, Jiang Y, Tao D (2020). Molecular detection of Anaplasma spp. in dairy cattle in southern Xinjiang. China. Vet Parasitol Reg Stud Reports.

[51] Yang B (2020). First molecular evidence of Anaplasma spp co-infection in stray dogs from Anhui. China. Acta Trop.

[52] Zhang Y, Lv Y, Cui Y (2016). First molecular evidence for the presence of Anaplasma DNA in milk from sheep and goats in China. Parasitol Res.

[53] Barlough JE (1996). Nested polymerase chain reaction for detection of Ehrlichia equi genomic DNA in horses and ticks ( Ixodes pacificus ). Vet Parasitol.

[54] Barlough J (1996). Nested polymerase chain reaction for detection of *Ehrlichia equi* genomic DNA in horses and ticks (*Ixodes pacifcus*). Vet Parasitol.

[55] Kawahara M, Rikihisa Y, Lin Q (2006). Novel genetic variants of Anaplasma phagocytophilum, Anaplasma bovis, Anaplasma centrale, and a novel Ehrlichia sp. in wild deer and ticks on two major islands in Japan. Applied Environ Microbiol.

[56] de la Fuente J, Kocan KM (2006). Strategies for development of vaccines for control of ixodid tick species. Parasite Immunol.

[57] Yang J (2016). Molecular survey and characterization of a novel Anaplasma species closely related to Anaplasma capra in ticks, northwestern China. Parasit Vectors.

[58] Liu Z, Ma M, Wang Z (2012). Molecular survey and genetic identification of Anaplasma species in goats from central and southern China. Appl Environ Microbiol.

[59] WenPing Guo (2020). Extensive genetic diversity of Anaplasma bovis in ruminants in Xi'an. China. Ticks Tick Borne Dis.

[60] Li Y (2016). Molecular Survey of Anaplasma and Ehrlichia of Red Deer and Sika Deer in Gansu, China in 2013. Transbound Emerg Dis.

[61] Yang J, Han R, Niu Q (2018). Occurrence of four Anaplasma species with veterinary and public health significance in sheep, northwestern China. Ticks Tick Borne Dis.

[62] Yang J, Liu Z, Niu Q (2017). Molecular Detection of Anaplasma phagocytophilum in Wild Cervids and Hares in China[J]. Wildl Dis.

[63] Noaman V (2020). Epidemiological study on Anaplasma phagocytophilum in cattle: Molecular prevalence and risk factors assessment in different ecological zones in Iran. Prev Vet Med.

[64] Koh FX (2018). Molecular investigation of Anaplasma spp in domestic and wildlife animals in Peninsular Malaysia. Vet Parasitol Reg Stud Rep.

[65] Fernandes SJ, Matos CA, Freschi CR (2019). Diversity of Anaplasma species in cattle in Mozambique. Ticks Tick Borne Dis.

[66] Rjeibi MR, Ayadi O, Rekik M (2018). Molecular survey and genetic characterization of Anaplasma centrale, A. marginale and A. bovis in cattle from Algeria. Transbound Emerg Dis.

[67] Belkahia H, Ben SM, El MN (2017). Seasonal dynamics, spatial distribution and genetic analysis of Anaplasma species infecting small ruminants from Northern Tunisia. Infect Genet Evol.

[68] Dumler JS (2012). The biological basis of severe outcomes in Anaplasma phagocytophilum infection[J]. FEMS Immunol Med Microbiol.

[69] Cui Y, Yan Y, Wang X, Cao S, Zhang Y, Jian F, Zhang L, Wang R, Shi K, Ning C (2017). First molecular evidence of mixed infections of Anaplasma species in dogs in Henan. China Ticks Tick Dis.

[70] Ge Y, Yin H, Rikihisa Y, Pan W, Yin H (2016). Molecular detection of tick-borne rickettsiales in goats and sheep from Southeastern China. Vector Borne Zoonotic Dis.

[71] Yang J, Li Y, Liu Z, Liu J, Niu Q, Ren Q, Chen Z, Guan G, Luo J, Yin H (2015). Molecular detection and characterization of Anaplasma spp in sheep and cattle from Xinjiang, northwest China. Parasit Vectors.

[72] Zhang Y, Lv Y, Zhang F, Zhang W, Wang J, Cui Y, Wang R, Jian F, Zhang L, Ning C (2016). Molecular and phylogenetic analysis of Anaplasma spp. in sheep and goats from six provinces of China. J Vet Sci.

[73] Azmat M, Ijaz M, Farooqi S, Ghaffar A, Ali A, Masud A, Saleem S, Rehman A, Ali M, Mehmood K (2018). Molecular epidemiology, associated risk factors, and phylogenetic analysis of anaplasmosis in camel. Microb Pathog.

[74] Chochlakis D, Koliou M, Ioannou I (2009). Kawasaki disease and Anaplasma sp infection of an infant in Cyprus. Int J Infect Dis.

[75] Rar V, Tkachev S, Tikunova N (2021). Genetic diversity of Anaplasma bacteria: Twenty years later. Infect Genet Evol.

[76] Altay K, Erol U, Sahin OF (2022). The detection and phylogenetic analysis of Anaplasma phagocytophilum-like 1, A. ovis and A. capra in sheep: A capra divides into two genogroups. Vet Res Commun.

